# CDC6 Inhibits CDK1 Activity in MII-Arrested Oocyte Cell-Free Extract

**DOI:** 10.3390/ijms26094309

**Published:** 2025-05-01

**Authors:** Louis Dillac, Klaudia Porębska, Malgorzata Kloc, Rafal P. Piprek, Jean-Pierre Tassan, Jacek Z. Kubiak

**Affiliations:** 1 Dynamics and Mechanics of Epithelia Group, Institute of Genetics and Development of Rennes (IGDR), National Centre for Scientific Research (CNRS), Faculty of Medicine, University of Rennes, UMR 6290, 35043 Rennes, France; louis.dillac@gmail.com (L.D.); jean-pierre.tassan@univ-rennes1.fr (J.-P.T.); 2 Department of Molecular and Cellular Biology, University of Arizona, Tucson, AZ 85721, USA; 3 Laboratory of Molecular Oncology and Innovative Therapies, Military Institute of Medicine—National Research Institute, Szaserow 128, 04-141 Warszawa, Poland; kporebska@wim.mil.pl; 4 The Houston Methodist Research Institute, Houston, TX 77030, USA; mkloc@houstonmethodist.org; 5 Department of Surgery, The Houston Methodist Hospital, Houston, TX 77030, USA; 6 Department of Genetics, MD Anderson Cancer Center, The University of Texas, Houston, TX 77030, USA; 7 Department of Comparative Anatomy, Institute of Zoology and Biomedical Research, Faculty of Biology, Jagiellonian University, 30-387 Krakow, Poland; rafal.piprek@uj.edu.pl

**Keywords:** cell division cycle 6 (CDC6), cyclin-dependent kinase 1 (CDK1), cell division cycle 27 (CDC27), histone H1 kinase, cell cycle, metaphase II (MII) arrest, meiotic-to-mitotic transition, oocyte, oocyte activation, calcium, embryo, *Xenopus laevis*

## Abstract

The control of cyclin-dependent kinase 1 (CDK1) kinase activity is crucial for cell cycle progression. Cell division cycle 6 (CDC6) inhibits this activity in embryonic mitoses, and thus regulates the timing of cell division progression. The meiotic cell cycle differs greatly from the mitotic one. Metaphase II (MII)-arrested oocytes remain in prolonged M-phase state due to the high activity of CDK1 in the presence of CytoStatic Factor (CSF). The role of CDC6 in the control of CDK1 during MII and oocyte activation remains unknown. Here, we studied the role of CDC6/CDK1 interactions in *Xenopus laevis* cell-free extracts arrested in MII (CSF extract) and upon calcium activation leading to meiotic-to-mitotic transition. The CSF extract allows analysis of biochemical processes based on immunodepletion of selected proteins and facilitates manipulations using addition of recombinant proteins. We show by glutathione S-transferase (GST)-CDC6 pull-down that CDC6 associates with CDK1 in CSF extract and by histone H1 kinase assay that it downregulates CDK1 activity. Thus, CDC6-dependent inhibition of CDK1 is involved in the homeostasis of the MII-arrest. Upon CSF extract activation with calcium exogenous GST-CDC6 provokes accelerated transition from MII to interphase, while the depletion of endogenous CDC6 results in a slower transition to interphase. We demonstrate this by following both the phosphorylation state of CDK1 substrate cell division cycle 27 (CDC27) and histone H1 kinase assay. Importantly, increasing doses of GST-CDC6 proportionally accelerate CDK1 inactivation showing that CDC6 controls the dynamics of MII to interphase transition in a dose-dependent manner. Thus, CDC6 is a CDK1 silencer acting upon both the MII arrest and CSF extract activation by assuring the physiological activity of CDK1 during this meiotic arrest and correct timely inactivation of this kinase during the second process. Thus, we show that CDC6 controls CDK1 not only during mitotic divisions, but also in MII-arrest and the meiotic-to-mitotic transition in *Xenopus laevis* cell-free extracts. This study aims to bridge that gap by investigating CDC6 function using a biochemically controlled system.

## 1. Introduction

Beyond its established function in replication origin licensing, cell division cycle 6 (CDC6) has emerged as a key modulator of cyclin-dependent kinase 1 (CDK1) activity embryonic mitoses [1,2]. Specifically, we demonstrated that in *Xenopus* cycling extracts and mouse early embryos, CDC6 inhibits CDK1 kinase activity and delays the onset of mitosis, leading to the precise control of the timing of cell cycle transitions. CDC6 is also necessary for the physiological diauxic dynamics of CDK1 activation upon mitotic entry. This dual function in DNA replication and mitotic advancement highlights its role in ensuring the integrity and proper cell division progressing with physiological dynamics.

Following its role in DNA replication, the tight coordination between the timing of the cell cycle progression and the genetic program ensures the smooth progress of embryo development [3]. It begins upon the oocyte-to-embryo transition when metaphase II (MII)-arrested oocyte undergoes activation for development and enters the first interphase of the embryonic mitotic cell cycle. The timing of the cell cycle during both meiotic and mitotic divisions is largely regulated by complex interactions between kinases and phosphatases that control the phosphorylation state of key proteins. CDK1 is a key regulator of the entry and exit from the M phase, and thus is critical for the timing of the transition between successive cell cycles [4]. The MII-to-interphase transition in *Xenopus* oocytes is known to occur very rapidly, involving swift inactivation of histone H1 kinase, dephosphorylation of CDK1 substrates, and an important modification of CDK1-associated protein complex [3].

In early embryo development, our findings show CDC6 plays a vital role in controlling CDK1 activity in *Xenopus laevis* cell-free embryo extracts [2]. It also regulates the oocyte maturation and the first meiotic division [5]. The oocyte MII arrest and the second meiotic division triggered by fertilization or parthenogenetic oocyte activation are very particular stages requiring specific cell cycle control. Thus, it is not clear to what extent CDC6 is involved in MII-arrest and transition from meiotic to mitotic cell cycle upon oocyte activation.

CDC6 acts differentially on cyclin A- and cyclin B-associated CDK1 [1]. Although CDC6 is not a canonical CDK inhibitor, it has been shown to modulate CDK1 activity indirectly through interaction with regulatory proteins. We have shown recently that the *Xenopus* cyclin-dependent kinase (CDK) inhibitor Xic1 associates with both CDC6 and CDK1 in a cell cycle-dependent manner and may serve as an intermediate factor between CDC6 and CDK1/cyclin B. It is likely that multiple, non-mutually exclusive mechanisms contribute to CDC6-dependent inhibition of CDK1, including the involvement of potential other CDK1 inhibitors.

Matured oocytes ready for fertilization are arrested in meiotic metaphase II with high CDK1 activity stabilized by CytoStatic Factor (CSF) [3]. MII-arrest is a highly dynamic state from the molecular point of view. Cyclin B is constantly proteolytically degraded and synthesized, and the balance between these two processes regulates the level of CDK1 activity. An important shift in the composition of protein complex around CDK1 occurs indicating that other proteins playing a part in CDK1 inactivation and its subsequent interphasic functions may take place [3]. As the CDC6-dependent mechanism downregulates CDK1 activity in embryonic mitoses, we asked in the current study if it is also involved in CDK1 regulation in MII-arrested oocytes and upon their activation. Notably, similar regulatory mechanisms have been observed in mammalian oocytes, as suggested in previous studies using chemical inhibitors of CDK1 [6,7]. By comparing our findings in *Xenopus* cell-free extracts and in living mouse embryos [2] with the latter CDK1 inhibition in various mammalian oocytes, we highlight the broader relevance of CDC6 across species and in vivo vs. in vitro studies in cell-free extracts.

## 2. Results

### 2.1. Cell Division Cycle 6 (CDC6) Interacts with Cyclin-Dependent Kinase 1 (CDK1) in CytoStatic Factor (CSF) Extract and upon Calcium Addition During Meiotic-to-Mitotic Cell Cycle Transition

**Figure 1:**
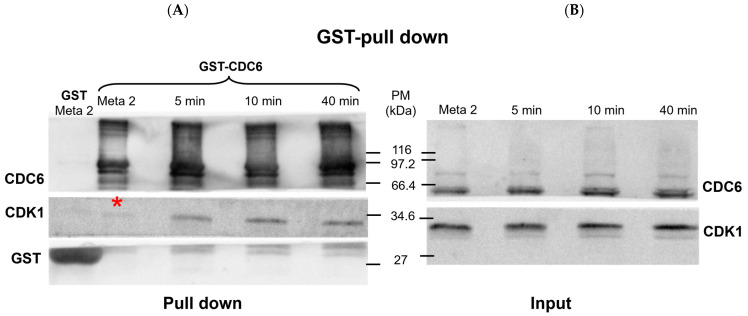
Cell division cycle 6 (CDC6) interacts with and inhibits cyclin-dependent kinase 1 (CDK1) during the metaphase II (MII)-to-interphase transition. (**A**) Western blot showing glutathione S-transferase (GST) pull-down performed on *Xenopus laevis* oocyte extracts in metaphase II and at 5, 10, or 40 min after calcium activation. GST pull-down was conducted using soluble bacterial fraction expressing either GST-CDC6 or GST alone, incubated with oocyte extracts. Samples were precipitated using glutathione Sepharose beads. GST-CDC6, but not GST alone, associates with CDK1, as detected with anti-PSTAIR antibodies. (**B**) Input lanes confirm presence of endogenous CDC6 and CDK1 at each timepoint. These inputs correspond to oocyte extract samples taken before GST pull-down. Both blots (**A**,**B**) were run on 12% polyacrylamide gels and transferred onto membranes for Western blotting. Membranes were probed using antibodies against GST, CDC6, and PSTAIR (to detect CDK1). PM = protein marker (molecular weight standard). Timepoints indicate minutes post-calcium addition. Note: Red asterisk denotes specific band corresponding to CDC6-CDK1 interaction.

First, we checked if there is an interaction between CDC6 and CDK1 in CSF extract and upon its activation with calcium ions, using glutathione S-transferase (GST) pull-down assays (Figure 1).

The Western blot in Figure 1 shows the presence of CDC6 and CDK1 in the input and GST pull-down fractions. The lanes representing the input confirm the expression level of CDK1 and CDC6 in the *Xenopus* CytoStatic Factor (CSF) extract at metaphase II (MII), as well as at 5, 10, and 40 minutes after calcium addition.

As shown in the pull-down lanes (Figure 1A), GST-CDC6 effectively pulls down a slight quantity of CDK1 in the CSF extract, which confirms an interaction between CDC6 and this kinase during the MII arrest. At 5 min post-activation, the CDK1 pulled down with GST-CDC6 increases and it becomes more abundant at 10 min post-activation. A similar quantity of CDK1 associated with GST-CDC6 is detected at 40 min post-activation. The GST pull-down control (GST lanes) shows no detectable CDK1 signal, which confirms the specificity of the CDK1–CDC6 interaction observed in the GST-CDC6 pull-down. Thus, the positive CDK1/CDC6 interaction shown by GST-CDC6 pull-down shows that it is maintained during MII arrest and increased following the CSF extract activation.

### 2.2. Exogenous Glutathione S-Transferase–Tagged Cell Division Cycle 6 (GST-CDC6) Inhibits Cyclin-Dependent Kinase 1 (CDK1) Activity in Xenopus CytoStatic Factor (CSF) Extracts

**Figure 2:**
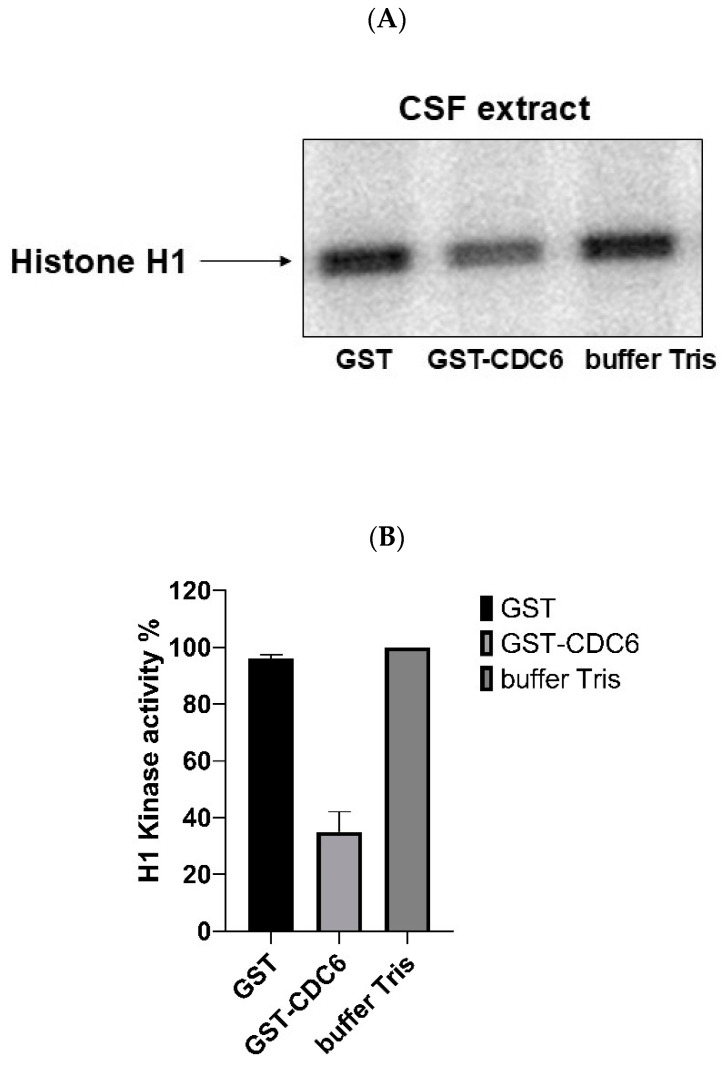
Exogenous glutathione S-transferase–tagged cell division cycle 6 (GST-CDC6) inhibits cyclin-dependent kinase 1 (CDK1) activity in *Xenopus laevis* CytoStatic Factor (CSF) extracts. (**A**) Representative autoradiograph showing histone H1 phosphorylation as a readout of CDK1 activity in extracts treated with buffer (control), glutathione S-transferase (GST) alone, or GST-CDC6 (15 nM). Extracts were incubated in maturation-promoting factor (MPF) buffer containing 0.5 mM sodium orthovanadate and protease inhibitors, 0.4 mg/mL histone H1, 1 μCi [γ-³²P] ATP, and 0.8 mM ATP. Reactions were incubated for 30 min at 30 °C, then stopped by adding Laemmli buffer. Histone H1 kinase activity was assessed using 12% SDS-PAGE, followed by autoradiography to measure phosphorylation of histone H1 kinase activity in dried SDS-PAGE gel. (**B**) Quantification of histone H1 phosphorylation was performed by densitometry using ImageJ software (version 1.54i, National Institutes of Health, Bethesda, MD, USA; https://imagej.nih.gov/ij/) and normalized to control condition. A one-way ANOVA followed by post hoc multiple comparisons was used to assess statistical significance among the three conditions. Data are presented as mean ± standard deviation (SD) from *n* = 3 independent experiments. Statistical significance was set at *p* < 0.05.

To assess the role of CDC6 in modulating CDK1 activity, we performed histone H1 kinase assays using *Xenopus* CSF extracts. Histone H1 is a well-established substrate of CDK1, phosphorylated in vivo and in vitro, and commonly used in kinase assays. Figure 2A,B shows the results of the measurement of CDK1 kinase activity in the presence of added: GST, GST-CDC6, and only the buffer. The results reveal that in the GST control and buffer control lanes there is significant phosphorylation of Histone H1, indicating active CDK1 kinase. However, in the presence of GST-CDC6, the phosphorylation of histone H1 is significantly reduced. This demonstrates that GST-CDC6 inhibits CDK1 activity in CSF extract and suggests that the endogenous CDC6 present in oocytes could have a similar action towards CDK1 in metaphase II (MII)-arrested oocytes.

### 2.3. Depletion of Cell Division Cycle 6 (CDC6) from CytoStatic Factor (CSF) Extract Increases Cyclin-Dependent Kinase 1 (CDK1) Activity

**Figure 3:**
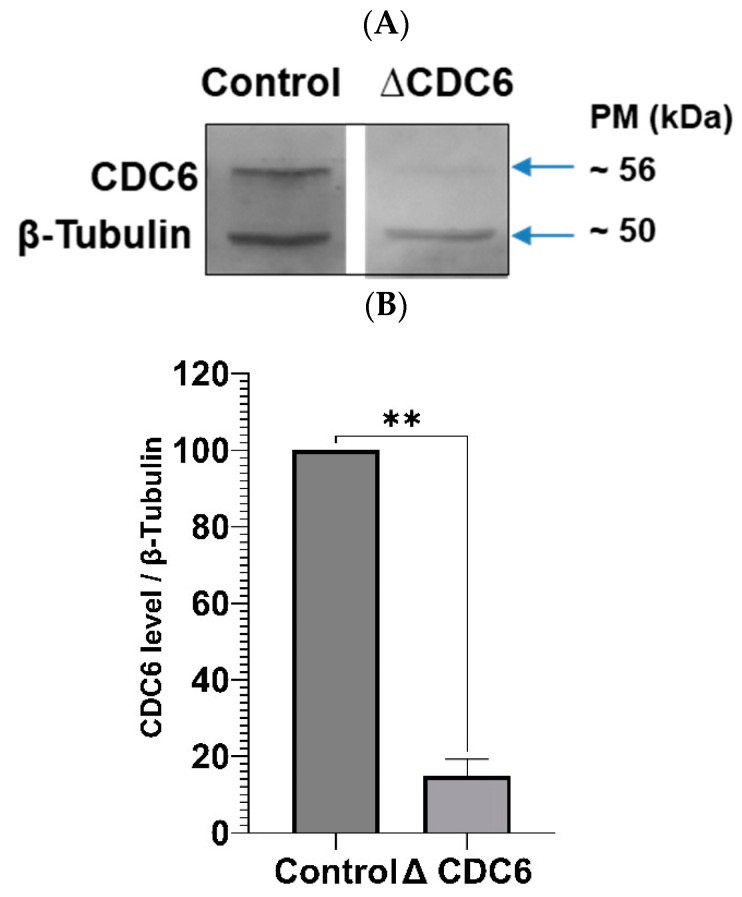

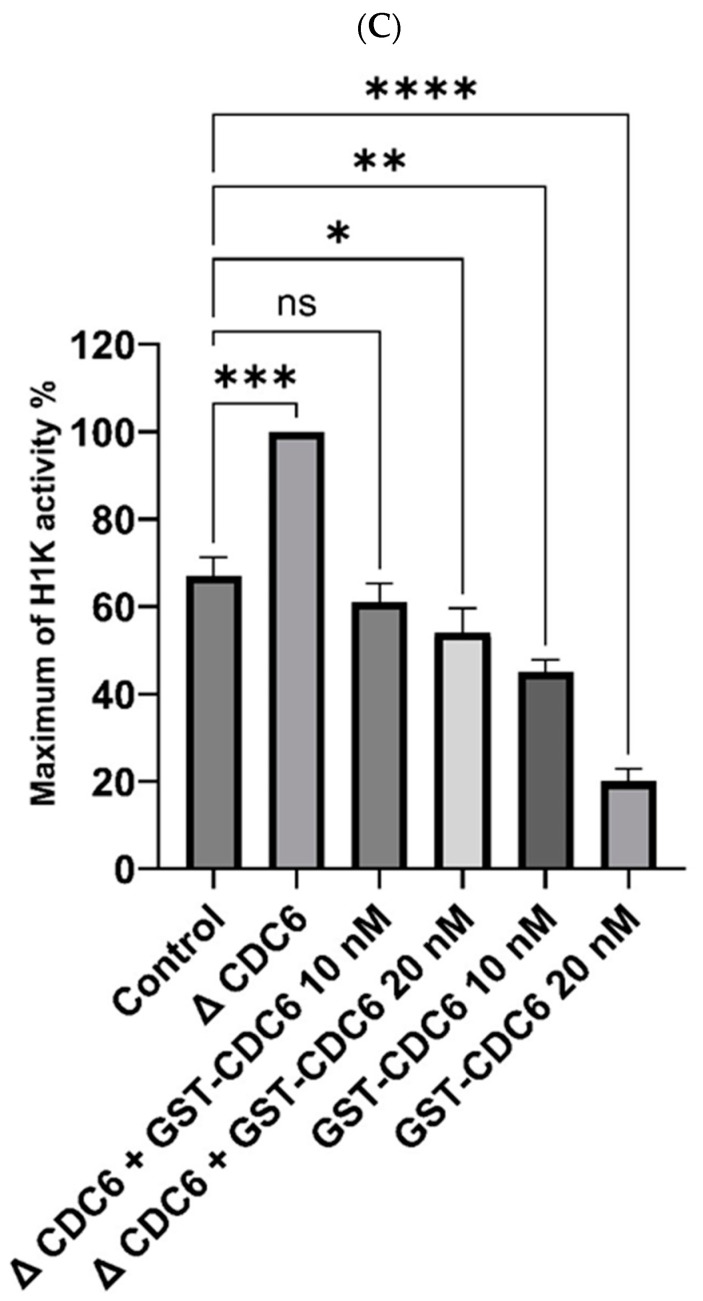
Depletion of cell division cycle 6 (CDC6) from CytoStatic Factor (CSF) extracts increases cyclin-dependent kinase 1 (CDK1) activity, while overexpression of CDC6 decreases CDK1 activity in a dose-dependent manner. (**A**) CDC6 was immunodepleted from *Xenopus laevis* CSF extracts using an anti-CDC6 antibody. CDC6 depletion was confirmed by Western blot. (**B**) Quantification of CDC6 levels was normalized to β-tubulin, which served as a loading control. An unpaired two-tailed t-test was used to compare control and CDC6-depleted conditions. (**C**) Extracts were diluted in maturation-promoting factor (MPF) buffer containing 0.5 mM sodium orthovanadate and protease inhibitors, 0.4 mg/mL histone H1, 1 μCi [γ-³²P] ATP, and 0.8 mM ATP. Reactions were then allowed to sit at 30 °C for 30 min and terminated by Laemmli buffer addition. Histone H1 phosphorylation was quantified as described above. A one-way ANOVA followed by post hoc multiple comparisons was used to assess statistical significance between conditions. In both panels, data are presented as mean ± standard deviation (SD) from *n* = 3 independent experiments. Statistical significance was set at *p* < 0.05 and is indicated as follows: * *p* < 0.05; ** *p* < 0.01; *** *p* < 0.001; **** *p* < 0.0001. Note: Although a visible gap appears between the two lanes in the Western blot, both lanes were run on the same gel. The image was digitally rearranged after flipping and cropping for clarity of presentation.

To verify if endogenous CDC6 inhibits CDK1 activity in CSF extract, we depleted CDC6 as described before [6] and measured CDK1 activity in a histone H1 assay. The efficiency of CDC6 depletion is shown in Figure 3A,B. As shown in Figure 3C, CDC6 depletion increases histone H1 activity in CSF extract by about 30%. Thus, endogenous CDC6 indeed downregulates CDK1 activity in the CSF extract.

Further, we analyzed whether exogenous GST-CDC6 may efficiently replace the endogenous one in CDC6-depleted CSF extract. As shown in Figure 3C the addition of glutathione S-transferase–tagged CDC6 (GST-CDC6) to CDC6-depleted extract significantly lowered histone H1 kinase activity in a dose-dependent manner: to about 40% less with 10 nM GST-CDC6 and 45% less with 20 nM GST-CDC6 (Figure 3C). Accordingly, 10 and 20 nM GST-CDC6 addition to the control CSF extract containing endogenous CDC6 resulted in further dose-dependent reduction of this activity as compared to histone H1 activity in the control extract (Figure 3C).

### 2.4. Cell Division Cycle 6 (CDC6) Accelerates Cell Division Cycle 27 (CDC27) Dephosphorylation upon CytoStatic Factor (CSF) Extract Activation

**Figure 4:**
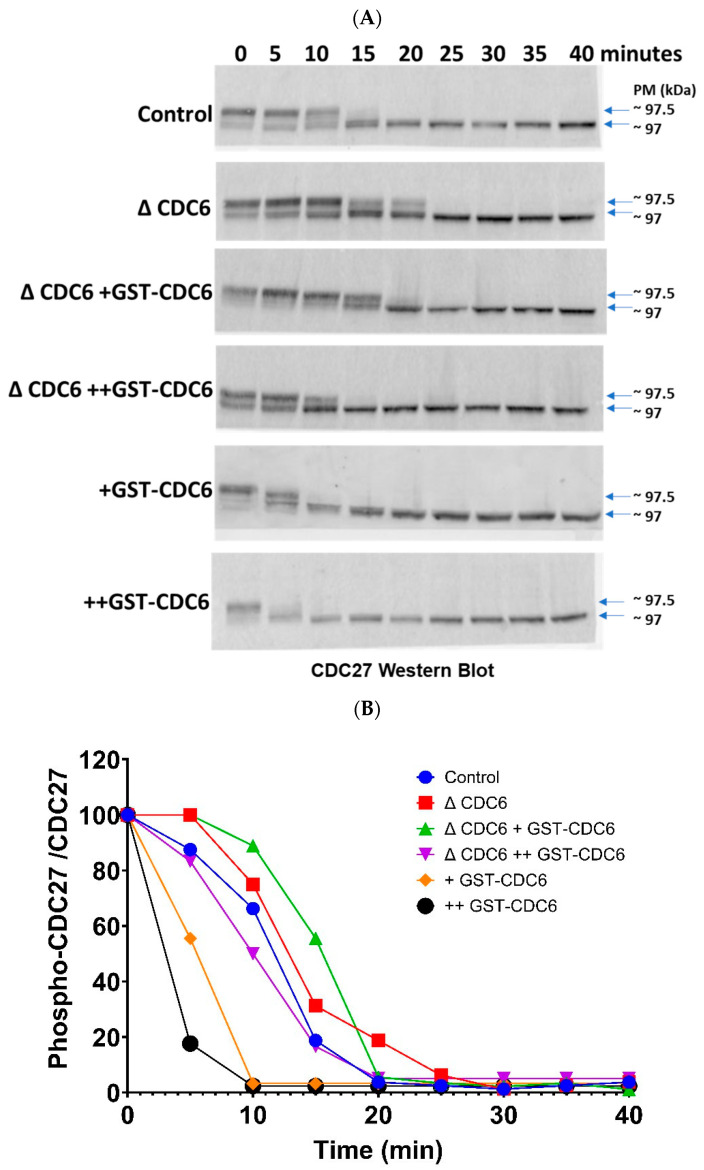
Cell division cycle 6 (CDC6) regulates cyclin-dependent kinase 1 (CDK1) inactivation dynamics during the metaphase II (MII)–to–interphase transition by modulating cell division cycle 27 (CDC27) phosphorylation. (**A**) Western blot analysis was performed to assess phosphorylation state of CDC27 in *Xenopus laevis* CSF extracts under six experimental conditions: (i) control (physiological CDC6), (ii) CDC6-depleted (ΔCDC6), (iii) ΔCDC6 + 10 nM glutathione S-transferase–tagged CDC6 (GST-CDC6) (+), (iv) ΔCDC6 + 20 nM GST-CDC6 (++), (v) control + 10 nM GST-CDC6 (+), and (vi) control + 20 nM GST-CDC6 (++). Samples were collected at multiple timepoints over a 40 min calcium-induced activation time course. CDC27 phosphorylation was inferred from electrophoretic mobility shifts, with the upper band representing the phosphorylated form. Molecular weight of CDC27 is approximately 95 kDa. Proteins were separated by 8% SDS-PAGE and transferred to nitrocellulose membranes. Membranes were blocked in PBS-T containing non-fat dry milk, incubated overnight at 4 °C with anti-CDC27 primary antibodies, and then incubated with alkaline phosphatase-conjugated secondary antibodies. Detection was performed using enhanced chemifluorescence (ECF) reagents. Timepoints represent minutes after calcium addition. Although a no-calcium control was not included in this experiment, previous studies have shown that CDK1 activity persists in CSF-arrested extracts in absence of calcium stimulation [8]. (**B**) Quantification of CDC27 phosphorylation (**B**) was performed by densitometry using ImageJ. The intensity of the phosphorylated CDC27 band (upper band) was normalized to the total CDC27 signal at each timepoint and plotted to show the dynamics of CDC27 dephosphorylation across conditions.

CDC27, a subunit of the anaphase-promoting complex/cyclosome (APC/C), is known to be a direct substrate of cyclin-dependent kinase 1 (CDK1) phosphorylation. Thus, the dynamics of CDC27 dephosphorylation reflect the dynamics of CDK1 inactivation. We thus examined the phosphorylation state of CDC27 in CSF extract and after its activation by calcium ions addition (Figure 4A,B).

In the control extract, CDC27 phosphorylation decreases progressively over the time course from 5 min to 20 min, when the whole pool of CDC27 is dephosphorylated. At 20 min post-activation, CDK1 inactivation is completed, no CDC27 is phosphorylated, and the extract is in interphase. In the CDC6-depleted extracts (ΔCDC6), we observed sustained CDC27 phosphorylation, and at 20 min post-activation an important part of the CDC27 band remains up-shifted. The total CDC27 dephosphorylation occurs at 25 min post-activation. This indicates that in the absence of CDC6, CDK1 remains active for a prolonged time, thus preventing the dephosphorylation of CDC27 within the frame of the control 20 min. As a result, the metaphase II (MII)–to–interphase transition is delayed, thus underscoring the importance of CDC6 in promoting CDK1 inactivation. When glutathione S-transferase–tagged CDC6 (GST-CDC6) was reintroduced into the CDC6-depleted extracts at 10 nM and 20 nM concentrations (ΔCDC6 +GST-CDC6 and ΔCDC6 ++GST-CDC6), the delay in CDC27 dephosphorylation was shortened and the total dephosphorylation occurs at 15 and 10 min post-activation for 10 and 20 nM GST-CDC6, respectively. The supplementation of the control extract with GST-CDC6 resulted in an additional acceleration of CDC27 dephosphorylation. It occurred between 10 and 15 min for 10 nM GST-CDC6 (+GST-CDC6) and between 5 and 10 min for 20 nM GST-CDC6 (++GST-CDC6).

These results show the importance of CDC6 in promoting CDC27 dephosphorylation upon CSF extract activation.

### 2.5. Cell Division Cycle 6 (CDC6) Ensures Timely Histone H1 Kinase Inactivation During the Metaphase MII–Interphase Transition

**Figure 5:**
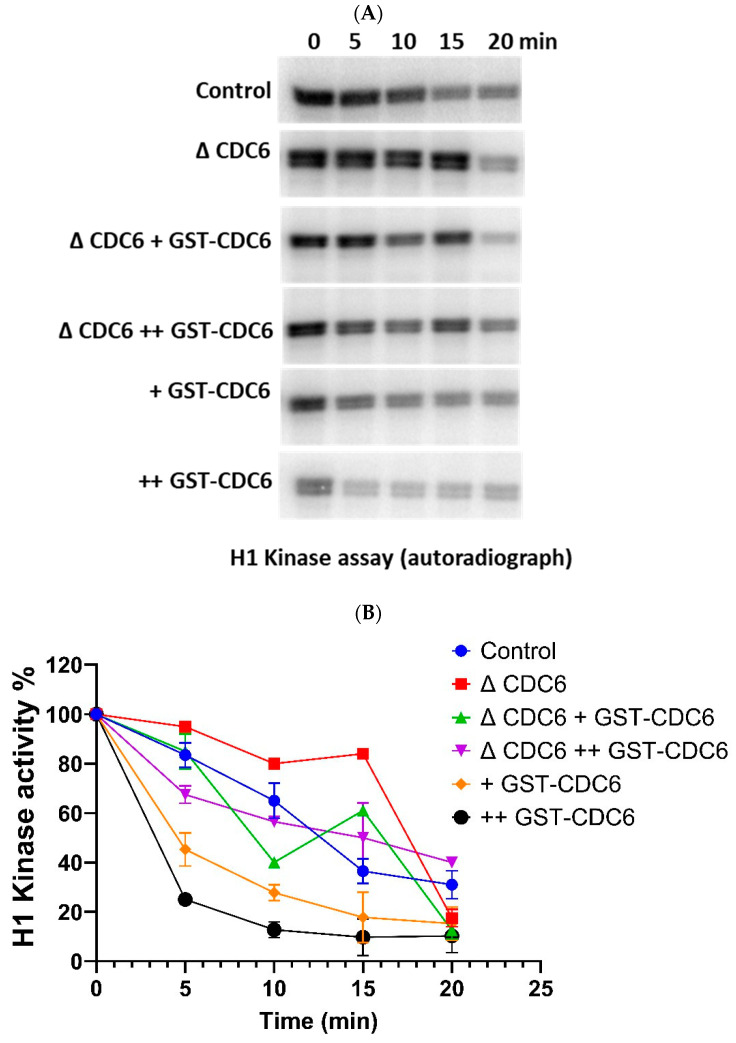
Cell division cycle 6 (CDC6) regulates the kinetics of cyclin-dependent kinase 1 (CDK1) inactivation during the metaphase II (MII)–to–interphase transition. (**A**) Representative autoradiograph showing histone H1 phosphorylation as a readout of CDK1 kinase activity in *Xenopus laevis* CSF extracts under six experimental conditions: (i) control (physiological CDC6), (ii) CDC6-depleted (ΔCDC6), (iii) ΔCDC6 + 10 nM glutathione S-transferase–tagged CDC6 (GST-CDC6) (+), (iv) ΔCDC6 + 20 nM GST-CDC6 (++), (v) control + 10 nM GST-CDC6 (+), and (vi) control + 20 nM GST-CDC6 (++). (**B**) Quantification of CDK1 activity based on histone H1 phosphorylation shown in (**A**). Quantification was performed as previously described. Statistical analysis was performed using two-way ANOVA. Treatment, time, and interaction effects were all highly significant (*p* < 0.0001).

Further, we explored the dynamics of cyclin-dependent kinase 1 (CDK1) activity over time in *Xenopus laevis* extracts during the MII–interphase transition, under the same experimental conditions, by measuring histone H1 kinase activity (Figure 5A,B).

In the control condition, at 5 min, CDK1 activity is still high, but it begins to decline sharply, becoming moderate by 10 min, and falling further to low levels by 15 min and very low by 20 min.

In the CDC6-depleted extract, CDK1 inactivation is markedly delayed. At 5 min, CDK1 remains highly active. Unlike the control, CDK1 activity in ΔCDC6 extracts declines only slightly by 10 min, and the decline continues at a much slower rate than in control, reaching low levels by 20 min post-activation comparable to 15 min time-point in the control extract. This delayed inactivation of CDK1 indicates a delay in the MII–interphase transition and further supports the hypothesis that CDC6 is essential for promoting CDK1 inactivation during this phase of the meiotic to mitotic cell cycle transition.

To rescue the CDC6-depleted phenotype, we reintroduced glutathione S-transferase–tagged CDC6 (GST-CDC6) at two different concentrations, 10 nM and 20 nM, and measured the effect on CDK1 activity. Both concentrations restored CDK1 inactivation dynamics more effectively than the control extract, with 20 nM GST-CDC6 showing slightly faster kinetics at early timepoints.

For comparison, we also measured CDK1 activity in extracts to which we added GST-CDC6 at 10 nM and 20 nM. In both cases, CDK1 activity declines much faster and in a dose-dependent manner, with 20 nM GST-CDC6 showing a faster rate of inactivation compared to 10 nM GST-CDC6 addition.

All these results confirm the critical role of CDC6 in promoting timely cell division cycle 27 (CDC27) dephosphorylation and histone H1 kinase inactivation during the MII–interphase transition, thus ensuring the proper timing during oocyte-to-embryo transition.

## 3. Discussion

Cell division cycle 6 (CDC6) inhibits cyclin-dependent kinase 1 (CDK1) activity, delaying mitotic entry and ensuring proper timing of cell cycle transitions. The inhibitory function of CDC6 towards CDK1 is likely mediated through specific cyclin docking motifs, as demonstrated in yeast models [9]. Our previous results and those presented in the current article suggest that a similar molecular mechanism may regulate CDC6-CDK1 interaction and CDC6-mediated CDK1 inhibition in *Xenopus laevis* mitotic and meiotic cell-free extracts. Our study provides novel insights into CDC6 inhibitory regulation of CDK1 activity in CytoStatic Factor (CSF) extract and during meiotic to mitotic transition upon CSF extract activation, aligning with our previous findings highlighting CDC6′s role in the control of embryonic mitotic divisions [1]. In contrast to its traditionally understood role in licensing DNA replication during S-phase, CDC6 has emerged as a key regulator of the M-phase both in maturing oocytes and in early embryos [5,10]. This dual functionality of CDC6 underscores its importance in the regulation of both S- and M-phase, and importantly both in meiotic and mitotic divisions. Our results presented in the current paper support these observations, pointing to the important role of CDC6 also during oocyte metaphase II (MII) arrest and the transition from oocyte to the embryo.

Our data show that MII–interphase transition in the activated CSF extract is accelerated by increased levels of CDC6 and proceeds slower in the absence of CDC6 or upon diminution of this protein in the extract before activation by calcium ions. As our glutathione S-transferase–tagged CDC6 (GST-CDC6) pull-down experiments show the increase of CDC6/CDK1 association in the protein complex upon CSF extract activation (see Figure 1), we hypothesize that CDC6 may actively participate in CDK1 inactivation during this period. Alternatively, a lower-than-physiological level of CDC6 in the CSF extract prior to activation (e.g., following CDC6 depletion), leads to elevated histone H1 kinase activity of CDK1 at the starting point (see Figure 3B). This higher initial activity may, in turn, prolong the time required for complete CDK1 inactivation. On the contrary, in the case of CDC6 addition to the CSF extract the starting point to inactivate histone H1 kinase is set at lower levels (see Figure 3B), and thus may shorten the time necessary to inactivate lower levels of histone H1 kinase activity. These findings support the idea that both the timing of CDC6 action and the initial CDK1 activity level contribute to the regulation of MII/interphase transition.

Previous yeast studies have revealed that cyclin-specific docking mechanisms, such as the LxF motif, regulate the interaction of Cdc6 with CDKs, adding a layer of complexity to how Cdc6 functions during mitotic exit [9]. This interaction, mediated through the phospho-adaptor Cks1 in yeast, may suggest that similar cyclin docking motifs are crucial for CDC6 interaction with CDK1 in vertebrates like *Xenopus*. Furthermore, human CDC6 has been identified as a mitotic substrate of Polo-like kinase 1 (PLK1), where phosphorylation at threonine 37 enhances its interaction with CDK1, resulting in inhibition of CDK1 activity and activation of separase to facilitate anaphase progression [11]. Thus, we suggest that similar molecular mechanisms may operate in *Xenopus* oocytes and embryos.

The paper by Calzada and colleagues (2001) suggests that yeast Cdc6 can cooperate with inhibitors such as Sic1 and Hct1 to directly inhibit the cyclin-dependent kinase Cdc28, the budding yeast (*Saccharomyces cerevisiae*) homolog of CDK1, thus promoting cyclin degradation and exit from the mitotic state [12]. Our previous and current findings add to this understanding by demonstrating that in *Xenopus laevis* cell-free extracts the GST-tagged CDC6 interacts with and inhibits the CDK1 activity, probably via inhibitory action of the *Xenopus* CDK inhibitor Xic1. This inhibitory action of the CDC6-dependent mechanism assures both the right timing of mitotic divisions and the physiological dynamics of CDK1 activation upon M-phase. In the current study, we show that GST-CDC6 accelerates the exit of the meiotic M-phase when added to calcium-activated CSF extracts, as evidenced by the acceleration of both cell division cycle 27 (CDC27) dephosphorylation and histone H1 kinase inactivation. These results strongly suggest that the CDC6-dependent mechanism also controls the CDK1 activity in MII-arrested oocytes and upon their activation. These findings highlight the regulatory role of CDC6 in oocyte activation. The CSF extract allows to analyze the biochemical mechanisms upon MII arrest in much easier way than in ovo, because its manipulations do not activate the extract, which often happen when proteins or drugs are injected to living MII oocytes. Moreover, calcium addition allows the activation of CSF extract, while fertilization or parthenogenetic activation of MII oocytes often activates only a fraction of them. Therefore, using this system allows to identify the molecular mechanisms easier than by in ovo studies. Despite these advantages, further in vivo studies are needed to determine how these mechanisms operate in whole oocytes and embryos.

## 4. Materials and Methods

### 4.1. Xenopus CytoStatic Factor (CSF) Extract Preparation 

Egg extracts were made from *Xenopus laevis* oocytes in metaphase II (MII) under CSF conditions, as reported previously [13]. The CSF extract is composed of isolated MII-arrested cytoplasm of oocytes and mirrors biochemical processes occurring during MII arrest [13]. Oocytes were placed in ultracentrifuge tubes and stored at 4° C in XB buffer containing 10 g/mL aprotinin, leupeptin, pepstatin, 0.1 mM AEBSF, and 25 g/mL cytochalasin D (all from MilliporeSigma, St. Louis, MO, USA). Eggs were crushed at 10,000× *g* for 10 min. The supernatant was further clarified by centrifugation at 10,000× *g* for 10 min at 4 °C. Samples were taken for analysis and stored at −80 °C. CSF extract was activated by calcium addition. Such an activated CSF extract mirrors the biochemical process occurring during MII oocytes parthenogenetic activation.

### 4.2. Production and Purification of Glutathione S-Transferase–Tagged Cell Division Cycle 6 (GST-CDC6)

CDC6 gene was introduced into pGEX-4T3 vector to make GST-tagged CDC6, as earlier reported [2]. The plasmid was introduced into *Escherichia coli* BL21 cells and cultured until the OD600 was 0.6–0.8. At this point, IPTG (1 mM) was added to induce GST-CDC6 expression for 4 h at 37 °C. The cells were harvested by spinning the cells 4000× *g* for 10 min at 4 °C, redissolved in lysis buffer (50 mM Tris-HCl, pH 7.5, 150 mM NaCl, 1 mm EDTA, 1% Triton X-100, 1 mm DTT, protease inhibitors) and lysed by sonication. The lysate was clarified by centrifugation at 12,000× *g* for 15 min at 4 °C. The fusion GST-CDC6 was purified by incubating the soluble lysate with glutathione Sepharose beads at 4 °C for 1 h with gentle rotation. The beads were washed three times with a wash buffer (50 mM Tris-HCl, pH 7.5, 150 mM NaCl, 0.1% Triton X-100). The GST-CDC6 was eluted by incubating the beads with elution buffer containing 50 mM Tris-HCl (pH 8.0) and 10 mM reduced glutathione at room temperature for 10 min. The eluted proteins were stored at −80 °C. All reagents were purchased from MilliporeSigma (St. Louis, MO, USA).

### 4.3. GST (Glutathione S-Transferase) Pull-Down Assay

The egg extracts were extracted by the process above. A volume of 500 μL of the extract was incubated at 4 °C for 1 h with GST–tagged cell division cycle 6 (GST-CDC6), or GST alone as a control. Then, 20 µL of glutathione Sepharose beads were added to the solution and left for an additional hour at 4 °C under light stirring. The beads were washed four times in wash solution (50 mM Tris-HCl, pH 7.5, 150 mM NaCl, 1% Triton X-100), and soluble proteins were eluted by boiling the beads in Laemmli buffer. All the samples were loaded on SDS PAGE gel and Western blotted for CDC6, then detected with PSTAIR antibody for CDK1. All reagents were purchased from MilliporeSigma (St. Louis, MO, USA). Western blotting was performed using equipment from Bio-Rad Laboratories (Hercules, CA, USA).

### 4.4. Immunodepletion of Cell Division Cycle 6 (CDC6)

Immunodepletion of CDC6 from *Xenopus* egg extracts was done using AffiPrep Protein A beads (Sigma, St. Louis, MO, USA) conjugated with anti-CDC6 antibodies or pre-immune serum (control) as previously discussed [2]. These beads were washed and incubated with pre-mitotic *Xenopus* embryo extracts at 4 °C. The process was repeated twice to eliminate CDC6 from the extract.

### 4.5. Western Blot Analysis

SDS-PAGE of proteins was carried out on nitrocellulose membranes (Hybond C, Amersham Biosciences, Amersham, UK) following Laemmli’s method [14]. The membranes were blocked in 5% non-fat dry milk in PBS-T (PBS containing 0.1% Tween-20) for an hour at room temperature, and overnight at 4 °C with primary antibodies including anti-GST, anti-CDC6, anti-CDK1, CDC27, and β-Tubulin. Afterward, the membranes were rinsed with PBS-T and incubated at room temperature for an hour with horseradish peroxidase (HRP) secondary antibodies or alkaline phosphatase secondary antibodies against rabbit or mouse.

We employed enhanced chemiluminescence (ECL) for HRP-labeled antibodies and chemifluorescence with the ECF reagent (Amersham Biosciences) for alkaline phosphatase-labeled antibodies. Signal quantification was performed with ImageQuant 5.2 software (Amersham Biosciences). 

All reagents were purchased from MilliporeSigma (St. Louis, MO, USA), unless otherwise stated. Western blotting was performed using equipment from Bio-Rad Laboratories (Hercules, CA, USA).

### 4.6. Histone H1 Kinase Activity Assay

Samples obtained from *Xenopus* egg extracts were diluted in maturation-promoting factor (MPF) buffer containing 0.5 mM sodium orthovanadate, protease inhibitors (leupeptin, aprotinin, pepstatin, chymostatin), 0.4 mg/mL histone H1, 1 μCi [γ-³²P] ATP, and 0.8 mM ATP. After 30 min of incubation at 30 °C, reactions were halted with Laemmli buffer and histone H1 kinase activity was determined by SDS-PAGE separation followed by autoradiography. Although 30 °C incubation for 30 min may seem extended, this condition reflects previous protocols using *Xenopus* extracts and allows consistent phosphorylation detection [2].

All reagents were purchased from MilliporeSigma (St. Louis, MO, USA). Western blotting was performed using equipment from Bio-Rad Laboratories (Hercules, CA, USA).

### 4.7. Statistical Analysis

All experiments were repeated at least twice to ensure reproducibility. Statistical analyses were performed using unpaired *t*-tests, one-way ANOVA with post hoc multiple comparisons, or two-way ANOVA where appropriate. Significance thresholds were set at *p* < 0.05, with additional significance levels indicated in the figures. Data are presented as mean ± standard deviation (SD).

## 5. Conclusions

In conclusion, cell division cycle 6 (CDC6) modulates both the timing of mitotic and meiotic divisions and the transition from the meiotic to mitotic cell cycle in cell-free extracts. We propose that the CDC6-dependent mechanism inhibits cyclin-dependent kinase 1 (CDK1) during all M-phases, regardless of their meiotic or mitotic character and plays a crucial role in the correct timing of M-phase events allowing the correct cell cycle progression. Thus, CDC6 plays an important role both during meiosis and mitosis in tuning the cell cycle clock, primarily dictated by cyclins accumulation and degradation cycle.

## Data Availability

The original contributions presented in this study are included in the article. Further inquiries can be directed to the corresponding author.

## Abbreviations

The following abbreviations are used in this manuscript:

MII	Metaphase II arrest
CSF	CytoStatic Factor
